# Photon-Assisted Perfect Conductivity Between Arrays of Two-Level Atoms

**DOI:** 10.1038/s41598-019-49606-y

**Published:** 2019-09-10

**Authors:** Chih-Chun Chang, Lee Lin, Guang-Yin Chen

**Affiliations:** Department of Physics, National Chung Hsing University, Taichung, 402 Taiwan

**Keywords:** Quantum optics, Single photons and quantum effects

## Abstract

We investigate interactions between two (parallel) arrays of two-level atoms (2LA) via photons through quantum electrodynamical interaction with one array (the source array) connected to a particle source, and we study the (photo-)resistivity of the other array (the measured array). The wave function of the interacted photon propagating in an array is a Bloch wave with a gap in its eigenvalue (the photonic dispersion). Due to interactions between arrayed 2LA and the dressed photonic field with non-linear dispersion, the conduction behaviors of the measured array can be very diversified according to the input energy of the particle source connected to the source array, and their relative positions. As a result, the resistivity of the measured array can be zero or negative, and can also be oscillatory with respect to the incoming energy of the particle source of the source array, and the separation between arrays.

## Introduction

The interactions between light and atoms and their diversified manifestations^1–7^ have been an important area of research in fundamental physics, and practical applications for many years. Among these researches, the phenomena of microwave-induced zero resistance (MIZR), and microwave-induced resistance oscillation (MIRO) in systems of two-dimensional electron gas (2DEG) have been studied by many researchers since their discoveries around 2002^1,4^. With the irradiation of microwave on these 2DEG samples, the (magneto-)resistance of the systems of semiconductor in two dimensions has oscillations^1–4,8–21^. And there have been many theories introduced to explain those phenomena^8,22–58^. In the above theoretical works, the displacement model and other related models^8,22–57^ with impurities required to participate in electron transportation are quite appropriate in systems with multiple impurities. On the other hand, as the concentration of impurities decreasing, the model of dressed photon with nonlinear dispersion^58^ seems to be a suitable theory. It is exhibited in ref.^58^ that MIZR & MIRO can arise due to interactions of quantum electrodynamics (QED) between photons and simple harmonic atoms in an array^59–63^ even in systems with no impurities. From the perspective of concentration of impurities, these two kinds of theories may be complimentary to each other in studying the propagation of electrons in systems exposed to photonic source.

**Figure 1:**
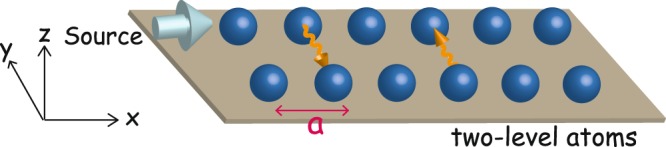
Model. An array of *N* two-level atoms with spacing *a* (*x*-direction) interacts with another parallel array of *N* two-level atoms connected with a particle source/sink through emitting and absorbing photons. Inter-array hopping of electrons is prohibited.

In this paper, we would report that resistance oscillation and zero resistance also appear in two parallel arrays of two-level atoms (2LA) (Fig. 1). We consider a model of two parallel arrays (*x*-direction) of 2LA each with *N* sites with no impurities. They can only interact with each other via emitting/absorbing photons through the QED coupling. Only one array (the source array) is connected to a source (sink) of electrons of the excited state; and we measure the resistivity of the other one (the measured array). The resistance of the measured array shows oscillations & zero-resistance with respect to the frequency of the external source and the separation between arrays. People might be reminiscent of the classical phenomenon of mutual induction between two loops with the potential (emf) along one loop influenced by the other loop by variation of magnetic flux. The way to achieve it can be the relative motion between the two loops, or the changing source of electric current for the magnetic field. Analogously, in our model, the resistance and therefore the electric potential difference across one array can be influenced by the other array by variation of the EM irradiation of photon through relative displacement between the two arrays and change of the source of electron. The experimental realizations of our model will be discussed later in the Discussion section.

Please be noted that a major difference between this model and that in ref.^58^ is that we also study the manifestations of the MIZR & MIRO effects with respect to the separation between parallel arrays. And we will show an interesting big decrease of (averaged) resistance between two parallel arrays with the increase of separation between them. In addition, the model of two-level systems discussed in this work is more related to potential applications to quantum memory and relevant systems. Furthermore, we will present a quantum analog of the potential of one EM set-up influenced by another one through photonic field interacting between them like the mutual-induction in the classical domain. From the point of view of accommodations of electrons, there is an essential difference between the atom of the two-level system and that of the simple-harmonic-oscillator (SHO) studied in ref.^58^. For a two-level atom, only one electron can be accommodated in an atom; while many electrons can be accommodated in an atom with eigenenergies of equal-energy-spacing like the SHO & the 2DEG in a magnetic field with Landau energy levels. Therefore, a two-dimensional array of SHO are more related to the 2DEG in a magnetic field.

## Results

In this paper, we study our system at temperature *T* = 0. The EM wave is assumed to be uniform along *y*, *z*-directions, and to move in $\hat{x}$. Therefore, in radiation gauge ($\nabla \cdot \overrightarrow{A}=0$), the vector potential $\overrightarrow{A}$ can be written as $\overrightarrow{A}(x)=(0,{A}_{y}(x),{A}_{z}(x))$. The photonic annihilation operator *A*, and the photonic creation operator *A*^†^ are defined as,

1 $$A=({A}_{y}+i{A}_{z})/\sqrt{2},\,{A}^{\dagger }=({A}_{y}-i{A}_{z})/\sqrt{2}.$$

Then the Hamiltonian for the free photon is

2 $${H}_{em}=\frac{1}{2}\,{\int }^{}\,dx\,[{\dot{\overrightarrow{A}}}^{2}+{(\nabla \times \overrightarrow{A})}^{2}]={\int }^{}\,dx\,({\dot{A}}^{\dagger }\dot{A}+\nabla {A}^{\dagger }\cdot \nabla A).$$

For an array of *N* 2LA, the Hamiltonian is

3 $$\begin{array}{rcl}{ {\mathcal H} }_{2LA}(\{c\}) & = & { {\mathcal H} }_{2LA}^{(2)}(\{c\})+{ {\mathcal H} }_{2LA}^{(4)}(\{c\}),\\ { {\mathcal H} }_{2LA}^{(2)}(\{c\}) & = & \sum _{\alpha =1,2}\,\sum _{j}\,({\varepsilon }_{\alpha }-i\delta ){c}_{\alpha ,j}^{\dagger }{c}_{\alpha ,j}+\frac{\lambda }{2}\,\sum _{\alpha ,\,j,\,\delta }\,[{c}_{\alpha ,\,j+\delta }^{\dagger }\,{c}_{\alpha ,j}+h.\,c.\,],\end{array}$$

4 $$\begin{array}{rcl} & = & \sum _{\alpha ,p}\,({\nu }_{\alpha }(p)-i\delta )\,{c}_{\alpha ,p}^{\dagger }\,{c}_{\alpha ,p},\end{array}$$

5 $$\begin{array}{rcl}{ {\mathcal H} }_{2LA}^{(4)}(\{c\}) & = & \frac{U}{2}\,\sum _{j}\,{c}_{2,j}^{\dagger }{c}_{1,j}^{\dagger }{c}_{2,j}{c}_{1,j}\\  & = & {{\mathscr{N}}}^{-1}\,\sum _{P,\,p,\,q}\,\frac{U}{2}\cdot {c}_{2,P/2+q}^{\dagger }\,{c}_{1,P/2-q}^{\dagger }\,{c}_{2,P/2+p}\,{c}_{1,P/2-p},\end{array}$$

6 $${\rm{where}}\,{\nu }_{\alpha }(p)={\varepsilon }_{\alpha }+\epsilon (p)\,(\alpha =1,2),\,{\rm{and}}\,\epsilon (p)=\lambda (1-\,\cos \,pa),$$

with *a* the lattice spacing, $\epsilon (p)$ the famous Bloch spectrum, and natural units is used $(c\equiv \hslash \equiv 1)$^64^. On the RHS of Eq. (3), the second term describes hoppings of electron from one site to its adjacent sites. Here the field operator of electron at site *j* on the upper (lower) energy level, the excited state (the ground state), is denoted as ${c}_{2,j}$ (${c}_{1,j}$) with ${\varepsilon }_{2}$ (${\varepsilon }_{1}$) the corresponding energy. And both the upper & lower fields can hop to their nearest-neighbor sites with $\frac{\lambda }{2}$ as the hopping coefficient. At low temperatures, there are two channels of conduction through transportations of particles of the upper field, and holes corresponding to vacancies of particles of the lower field. In Eq. (3), *δ* is small and positive. The Hamiltonian ${ {\mathcal H} }_{2LA}^{(4)}$ describes the on-site hard-core interaction between the upper & lower fields when *U* is set to $\infty$ to avoid the possibility of both the upper field particle and the lower field particle at the same site.

The interaction between the arrayed 2LA and photons is

7 $${ {\mathcal H} }_{{int}}(\{c\},A)=\sum _{j}\,g\,[A({x}_{j}){c}_{2,j}^{\dagger }{c}_{1,j}+h.\,c.\,].$$

And the Hamiltonian ${ {\mathcal H} }_{int}(\{c\},A)$ is the light-matter interaction adopted in QED in which a lower field can absorb a photon to become the upper field, and vice versa; the coupling constant $g\sim \sqrt{{e}^{2}/\hslash c}\sim 1/\sqrt{137}$ is the coupling between *bare* electrons and *bare* photons as is widely adopted in field theory literatures, *e*.*g*., ref.^64^. Please note that the collective behavior of this coupling constant between electrons and photons in different geometry or confinements results in different effective^65^ *coupling strength* in quantum optics.

### *t*-matrix

**Figure 2:**
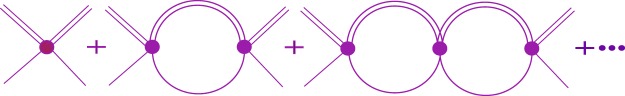
Ladder diagram. Diagrammatic expansion of $\langle q|\Gamma ({P}_{0},P)|p\rangle$ which is the sum of the repeated and continuous scatterings between the upper and the lower fields (ladder diagrams). The external legs are only for the eyes, the black dot “●” is *U* in Eq. (5), and the internal double-line (single line) represents the propagator of the upper (lower) field.

To handle the hard-core interaction ${ {\mathcal H} }_{2LA}^{(4)}$ (Eq. (5)), we can apply the method of binary collision which was developed in 1959^66–68^. We add up all the repeated scatterings between the upper and the lower field (*ladder* diagrams (Fig. 2)) to get a finite effective coupling $t(\lambda )$ between them at low energy.

We define $\langle q|\Gamma ({P}_{0},P)|p\rangle$ to be the sum of the ladder diagrams (in Fig. 2) which is the amplitude of repeated scatterings between upper field (1) and lower field (2) with incoming momenta *p*_1_ & *p*_2_, respectively $(p=\frac{1}{2}({p}_{1}-{p}_{2}))$, and outgoing momenta *q*_1_ & *q*_2_, respectively $(q=\frac{1}{2}({q}_{1}-{q}_{2}))$, and *P*_0_ is the total energy, *P* the total momentum^69^.

Following ref.^69^, we can obtain that

8 $$\langle q|\Gamma (P,{P}_{0})|p\rangle =2\,{{\bf{u}}}^{{\bf{T}}}(q)\cdot {\bf{K}}\,{[1+\frac{1}{2}{\bf{G}}(P,{P}_{0}){\bf{K}}]}^{-1}\cdot {\bf{u}}(p),$$

where

9 $$\begin{array}{ll} & {\bf{u}}(p)=[\begin{array}{c}1\\ \cos \,pa\end{array}],\,{\bf{K}}=[\begin{array}{cc}\frac{U}{2} & 0\\ 0 & 0\end{array}],\\  & {{\bf{G}}}_{\alpha \beta }(P,{P}_{0})\equiv \frac{4}{{\mathscr{N}}}\,\sum _{q}\,\frac{{u}_{\alpha }(q)\,{u}_{\beta }(q)}{{\nu }_{2,+}+{\nu }_{1,-}-{P}_{0}-i\delta },\\ {\rm{with}} & {\nu }_{\alpha ,\pm }={\nu }_{\alpha }(\frac{P}{2}\pm q),\,\alpha ,\beta =0,x.\end{array}$$

And by setting $U\to \infty$, we obtain the following equation,

10 $$\langle q|\Gamma (P,{P}_{0})|p\rangle =\frac{2}{{{\bf{G}}}_{00}(P,{P}_{0})};$$

that is, the hard-core interaction between the upper field particle and the lower field particle is equivalent to a soft-core one, and is independent of both the incoming & outgoing relative momenta (*p* & *q*). Thus, by the binary collision method^66,68,69^ (Eq. (8)), and Eqs (4) and (5), the following Hamiltonian can be obtained, and it is the low-energy soft-core effective Hamiltonian for the hard-core interaction,

11 $$\begin{array}{rcl}{H}_{eff}^{(4)}({c}^{\dagger },c) & = & \sum _{\alpha ,p}\,{\nu }_{\alpha }(p)\,{c}_{\alpha ,p}^{\dagger }\,{c}_{\alpha ,p}\\  &  & +\,\frac{1}{2{\mathscr{N}}}\,\sum _{P,\,p,\,q}\,{v}_{P}\,{c}_{2,P/2+q}^{\dagger }\,{c}_{1,P/2-q}^{\dagger }\,{c}_{2,P/2+p}\,{c}_{1,P/2-p},\end{array}$$

where

12 $${v}_{P}=t(P,\lambda )=\langle q|\Gamma (P,0)|p\rangle =\frac{2}{{{\bf{G}}}_{00}(P,0)}=\frac{\sqrt{{({\varepsilon }_{1}+{\varepsilon }_{2})}^{2}-{(2\lambda \cos \frac{P}{2})}^{2}}}{2},$$

is the effective new coupling used to replace the infinite *U* in ${ {\mathcal H} }_{2LA}^{(4)}(\{c\})$ (Eq. (5)). Please be noticed that the hard-core scatterings between upper & lower fields at low energy is summarized in *v*_*P*_ (Fig. 2).

### Electron propagator

**Figure 3:**
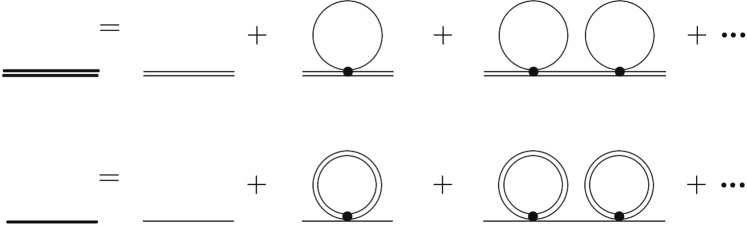
Diagrammatic expansions. Diagrammatic expansions of the renormalized propagators of the upper field ${\tilde{\Delta }}_{2}^{(1)}(k,\omega )$ (thick double-line) & the lower field ${\tilde{\Delta }}_{1}^{(1)}(k,\omega )$ (thick single-line) represented by the sum of free propagators of the upper field ${\tilde{\Delta }}_{2}^{(0)}(k,\omega )$ (thin double-line) & the lower field ${\tilde{\Delta }}_{1}^{(0)}(k,\omega )$ (thin single-line). The black dot “●” is the effective coupling *v*_*P*_ (Eq. (12)).

The renormalized propagators of the lower & upper fields ${\tilde{\Delta }}_{\alpha }^{(1)}(k,\omega )$ ($\alpha =1,2$) (with hard-core interaction taken into account) are diagrammatically represented in Fig. 3, and they satisfy the following Dyson’s equation (by Eqs. (3) and (9)),

13 $${\tilde{\Delta }}_{\alpha }^{(1)\pm }{(k,\omega )}^{-1}={[\frac{i}{\omega -{\nu }_{\alpha }(k)\pm i\delta }]}^{-1}-\frac{{\Sigma }_{\alpha }^{(1)}\,(k,\omega )}{i},$$

where the + sign is for particle propagator going forward in time, and the − sign is for propagator going backward in time (hole), and the effective mass ${\Sigma }_{\alpha }^{(1)}\,(k,\omega )$ is14

That is, the renormalized energy levels of both the upper & lower fields are shifted by ${\Sigma }_{1}^{(1)}\,(0,0)$ due to hard-core interaction. And these two effective masses can be well incorporated into our theory by redefining the energy levels to be ${\varepsilon ^{\prime} }_{\alpha }={\varepsilon }_{\alpha }+{\Sigma }_{1}^{(1)}\,(0,0)$. It follows that the energy difference (resonance energy of the 2LA)

15 $$\nu \equiv {\varepsilon }_{2}-{\varepsilon }_{1}={\varepsilon ^{\prime} }_{2}-{\varepsilon ^{\prime} }_{1}$$

is unchanged. In the mean time, the renormalized propagators of the upper & lower fields become

16 $${\tilde{\Delta }}_{\alpha }^{(1)\pm }(k,\omega )=\frac{i}{\omega -{\nu ^{\prime} }_{\alpha }(k)\pm i\delta },\,{\rm{with}}\,{\nu ^{\prime} }_{\alpha }(p)={\varepsilon ^{\prime} }_{\alpha }+\varepsilon (p),\,\alpha =1,2.$$

It should be remarked that the *renormalized* propagators of the *α* field (*α* = 2 for the upper field, & 1 for the lower) ${\tilde{\Delta }}_{\alpha }^{(1)\pm }(k,\omega )$ is the Fourier transform of the time-evolution amplitude $\langle {\tilde{\Psi }}_{\alpha }|{\mathscr{U}}(t)|{\tilde{\Psi }}_{\alpha }\rangle$ of the renormalized eigenstate corresponding to the *α* field $|{\tilde{\Psi }}_{\alpha }\rangle$ which carries the information of the hard-core interaction (or equivalently, the effective soft-core interaction) between the *bare* upper & lower fields, and ${\mathscr{U}}(t)$ is the time-evolution operator. Here, the renormalized eigenkets $|{\tilde{\Psi }}_{1}\rangle$ & $|{\tilde{\Psi }}_{2}\rangle$ are orthogonal to each other, and they form a basis of eigenkets that diagonalizes the Hamiltonian ${ {\mathcal H} }_{2LA}^{(2)}(\{c\})+{H}_{eff}^{(4)}({c}^{\dagger },c)$.

### Photon propagator

**Figure 4:**
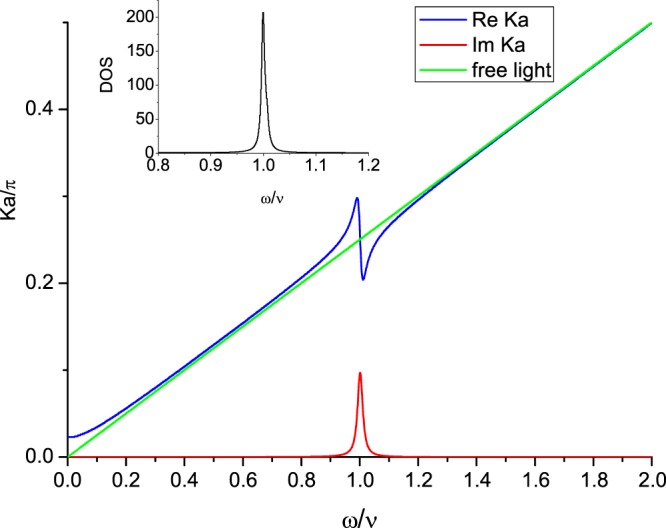
Dispersion. Dispersion relations of ${k}_{\omega }a/\pi (\,\equiv \,{\rm{Re}}\,{K}_{\omega }a/\pi )$ versus $\omega /\nu$ [blue line], and ${\kappa }_{\omega }a/\pi (\,\equiv \,{\rm{Im}}\,{K}_{\omega }a/\pi )$ versus $\omega /\nu$ [red line] of photonic field propagating in an array of 2LA. The green line represents the dispersion relation for free photon. Here we choose ${g}^{2}/a=1/125$, $\nu =\pi /4$, $\lambda =0.088\,\nu$, $\delta =\nu /100$. Being satisfying a similar (but not exactly the same) equation (Eq. (19)) with different parameters, the main figure looks like that of Fig. 2 in ref. ^71^. The inset shows the DOS (in arbitrary unit) of the (dressed) photon around the resonant energy $\nu$.

In this model, the lattice spacing will be denoted as *a*. Following ref.^70^, the photonic propagator $\tilde{G}(k^{\prime} ,\omega ^{\prime} ;k,\omega )$ satisfies the following Dyson’s equation

17 $$\begin{array}{rcl}\tilde{G}{(k^{\prime} ,\omega ^{\prime} ;k,\omega )}^{-1} & = & \tilde{G}{(k+h,\omega ^{\prime} ;k,\omega ^{\prime} )}^{-1}\delta (\omega -\omega ^{\prime} ){\delta }_{k+h,k^{\prime} }\\  & = & [{\tilde{G}}_{0}{(k^{\prime} ,\omega ^{\prime} )}^{-1}{\delta }_{k,k^{\prime} }+i\,\Pi (k^{\prime} ,\omega ^{\prime} ){\delta }_{k+h,k^{\prime} }]\cdot \delta (\omega -\omega ^{\prime} ),\end{array}$$

where ${\tilde{G}}_{0}(k^{\prime} ,\omega ^{\prime} )=i/(\omega {^{\prime} }^{2}-k{^{\prime} }^{2}+i\epsilon )$ is the propagator of free photon with $\epsilon$ being an infinitesimal positive number, $h=2n\pi /a$ is the reciprocal lattice vector (for all integer values of *n*), and $\Pi (k,\omega )$ is

18 $$\begin{array}{rcl}\Pi (k,\omega ) & = & \frac{i{g}^{2}}{2}\,{\int }^{}\,\frac{d\omega ^{\prime} }{2\pi }\frac{dk^{\prime} }{2\pi }\,{\tilde{\Delta }}_{2}^{(+)}(k^{\prime} +k,\omega ^{\prime} +\omega )\,{\tilde{\Delta }}_{1}^{(-)}(k^{\prime} ,\omega ^{\prime} ),\\  & = & \frac{{g}^{2}}{2}\,{\int }^{}\,\frac{dk^{\prime} }{2\pi }\frac{1}{\omega -\nu -2\lambda \,\sin (ka/2)\,\sin (k^{\prime} a)+2i\delta }\\  & = & \frac{{g}^{2}}{2a}\frac{1}{\omega -\nu +2i\delta }\cdot \frac{1}{\sqrt{1-{[\frac{2\lambda \sin (ka/2)}{\omega -\nu +2i\delta }]}^{2}}}\end{array}$$

which is the renormalization correction of self-mass to the photonic propagator from the light-matter interaction. We can then obtain the photonic dispersion relation through a calculation similar to that done in refs^70^ &^71^ as,

19 $${a}^{2}\Pi (k,\omega )\cdot \frac{\sin \,\omega a}{\omega a}+\,\cos \,\omega a-\,\cos \,ka=0,$$

as is shown in Fig. 4. (Please notice that our self-mass $\Pi (k,\omega )$ (Eq. (18)) is not exactly the same as that in refs^70^ &^71^). It is nonlinear with an energy gap near the energy spacing $\nu$, around there the momenta are complex corresponding to attenuated light waves. By ref.^71^, the dressed photon propagator is

20 $$\tilde{G}(l,\omega ;k,\omega )=|F(k;{\omega }_{k}){|}^{2}\frac{i}{{\omega }^{2}-{\omega }_{k}^{2}+i\epsilon }\,{\bar{\delta }}_{l,k},$$

where ${\bar{\delta }}_{l,k}$ denotes crystal momentum conservation, *i*.*e*., ${\bar{\delta }}_{l,k}=1$, if $l=k+2n\pi /a$; ${\bar{\delta }}_{l,k}=0$, otherwise; and the function $F(k,{\omega }_{k})$ is defined as^71^

21 $$F(k,{\omega }_{k})=\langle k|{\Psi }_{k}\rangle =\frac{1}{2|\Pi (k,\omega )|}{[\frac{1}{a}{\int }_{0}^{a}dx|f(x;k,{\omega }_{k}){|}^{2}]}^{-1/2},$$

22 $$f(x;k,{\omega }_{k})=-\,\frac{a}{2{\tilde{\omega }}_{k}}\frac{{e}^{-i(a-\Delta x)k}\,\sinh (\Delta x\,{\tilde{\omega }}_{k})+{e}^{iak}\,\sinh \,[(a-\Delta x)\,{\tilde{\omega }}_{k}]}{\cos (a\,{\tilde{\omega }}_{k})-\,\cos (ak)},$$

and $\Delta x=-\,[\frac{x}{a}]a+x$ ([] is the Gauss notation), ${\tilde{\omega }}_{k}=\sqrt{{\omega }_{k}^{2}+i\epsilon }$.

The amplitude that a photon with momentum *k*_*x*_ emitted from the source array (*s*) and propagating to the measured array (*m*) (a distance *y* afar) with momentum ${k^{\prime} }_{x}$ after a time *t* evolution has its Fourier transform as

23 $${}_{m}\langle \gamma ({k^{\prime} }_{x})|\tilde{{\mathscr{U}}}(\omega )|\gamma ({k}_{x})\rangle _{s}={\int }_{-\infty }^{\infty }\,\frac{d{k}_{y}}{2\pi }\,{e}^{i{k}_{y}y}\,\tilde{G}({k^{\prime} }_{x},{k}_{y},\omega ;{k}_{x},{k}_{y},\omega ).$$

And its integration over *k*_*x*_ & ${k^{\prime} }_{x}$, denoted as $\tilde{\Lambda }(\omega ,y)$, is very important in the multi-varied features of the (photo-)resistance of the measured array,

24 $$\begin{array}{rcl}\tilde{\Lambda }(\omega ,y) & = & {\int }_{-\infty }^{\infty }\,\frac{d{k}_{x}}{2\pi }\frac{d{k^{\prime} }_{x}}{2\pi }{}_{m}\langle \gamma ({k^{\prime} }_{x})|\tilde{{\mathscr{U}}}(\omega )|\gamma ({k}_{x})\rangle _{s}\\  & = & {\int }_{-\infty }^{\infty }\,\frac{d{k^{\prime} }_{x}}{2\pi }\frac{d{k}_{x}}{2\pi }\frac{d{k}_{y}}{2\pi }\frac{{e}^{i{k}_{y}y}\,i|F({k^{\prime} }_{x};{\omega }_{{k^{\prime} }_{x}}){|}^{2}}{{\omega }^{2}-{\omega }_{{k}_{x}}^{2}-{k}_{y}^{2}+i\delta }\,{\bar{\delta }}_{{k^{\prime} }_{x},{k}_{x}}\\  & = & {\int }_{0}^{\infty }\,\frac{d{k}_{y}}{2\pi }\,\cos ({k}_{y}\,y)\frac{|F({k}_{{\omega }_{1}};{\omega }_{1}){|}^{2}}{{\omega }_{1}}{(\frac{\partial {\omega }_{{k}_{x}}}{\partial {k}_{x}a})}_{{k}_{x}={k}_{{\omega }_{1}}}^{-1};\,({\omega }_{1}\equiv \sqrt{{\omega }^{2}-{k}_{y}^{2}})\\  & = & {\int }_{0}^{\infty }\,\frac{d{k}_{y}}{2\pi }\,\cos ({k}_{y}\,y)\,\frac{{\tilde{\rho }}_{E}(\sqrt{{\omega }^{2}-{k}_{y}^{2}})}{{a}^{2}\sqrt{{\omega }^{2}-{k}_{y}^{2}}}\end{array}$$

where ${\tilde{\rho }}_{E}(\omega )$ is the photonic density of state (DOS)^71^ dominated by energies around the energy gap (Fig. 4).

**Figure 5:**
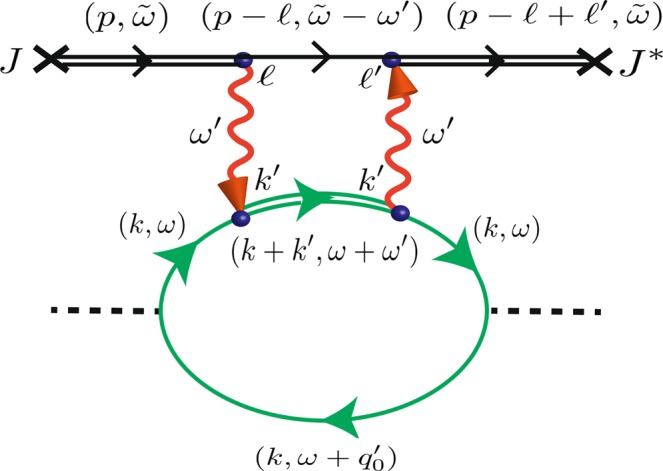
Diagram for the calculation of the conductivity. Diagram for the calculation of the conductivity (to the leading order) of an array of 2LA with the radiation emitted and absorbed by another parallel array which has an external source of electron with frequency ${\omega }_{e}$. The frequency is conserved, but the momentum satisfies only *crystal* momentum conservation. Each dotted line represents an insertion of momentum *k* from the current operator. The double-line (single-line) is the *renormalized* propagator of the upper field (lower field) ${\tilde{\Delta }}_{\alpha }^{(1)\pm }(k,\omega )$ (Eq. 16), and wavy-line represents the *renormalized* photonic propagator $\tilde{G}(k,\omega ^{\prime} ;k^{\prime} ,\omega ^{\prime} )$ (Eq. 17).

Without causing confusion, we shall depict in Fig. 5 the renormalized propagators of upper & lower fields of electron, and renormalized propagator of photon by thin double-line, single-line, and wavy-line, respectively, for brevity; and the term renormalized propagator will be simplified as propagator in the following paragraphs.

### DC conductivity

At zero temperature, the (renormalized) ground states $|{\tilde{\Psi }}_{1}\rangle$’s of the arrayed 2LA are occupied. Thus, the electron in the ground state can not transport unless it is raised to the (renormalized) excited state $|{\tilde{\Psi }}_{2}\rangle$, or its neighboring electrons are excited leaving holes there. Please be noted that the renormalized ground state is orthogonal to the renormalized excited state $\langle {\tilde{\Psi }}_{1}|{\tilde{\Psi }}_{2}\rangle =0$ (within the approximation of binary collision).

Before reaching the end, it is hard for electrons in the (renormalized) excited states in the measured array to drop to the (renormalized) ground states for being almost occupied. Therefore, it would be a good approximation to assume that electrons in the (renormalized) ground state $|{\tilde{\Psi }}_{1}\rangle$ of the measured array will not be excited to $|{\tilde{\Psi }}_{2}\rangle$ twice by absorbing and emitting and then absorbing again emitted photons from the source array during the process of transportation from one end to the other. It follows that we can calculate the Feynman diagram of interactions between the source array and the measured array (Fig. 5) to get the retarded current-current correlation $\langle j({q^{\prime} }_{0})j(\,-\,{q^{\prime} }_{0})\rangle$ to the leading order; then we obtain the DC conductivity through the Kubo formula,

25 $${\sigma }_{DC}=\mathop{\mathrm{lim}}\limits_{{q}_{0}\to 0}\,{\frac{-\,1}{{q}_{0}}\cdot {\rm{Im}}\langle j({q^{\prime} }_{0})j(\,-\,{q^{\prime} }_{0})\rangle ]}_{{q^{\prime} }_{0}=0}^{{q^{\prime} }_{0}={q}_{0}}={-\frac{\partial }{\partial {q}_{0}}{\rm{Im}}\langle j({q}_{0})j(-{q}_{0})\rangle |}_{{q}_{0}=0};$$

and the retarded current-current correlation corresponding to the Feynman diagram shown in Fig. 5 is

26 $$\begin{array}{l}{\langle j({q^{\prime} }_{0})j(-{q^{\prime} }_{0})\rangle }^{(P)}\\ \begin{array}{rcl} & = & {e}^{2}{\lambda }^{2}\,{\int }^{}\,\frac{d\tilde{\omega }}{2\pi }\frac{dp}{2\pi }\frac{d\omega ^{\prime} }{2\pi }\frac{dk^{\prime} }{2\pi }\frac{d\omega }{2\pi }\frac{dk}{2\pi }\frac{dl^{\prime} }{2\pi }\frac{dl}{2\pi }\,{k}^{2}\,{\Delta }_{1}^{R\,(+)}(k,\omega )\\  &  & \cdot \,{\tilde{J}}^{\ast }(p,\tilde{\omega }){\Delta }_{2}^{R\,(+)}(p+l^{\prime} -l,\tilde{\omega })\\  &  & \cdot \,ig\,{}_{s}\langle \gamma (l^{\prime} )|\tilde{{\mathscr{U}}}(\omega ^{\prime} )|\gamma (k^{\prime} )\rangle _{m}\,ig\\  &  & \cdot \,{\Delta }_{2}^{R\,(+)}(k+k^{\prime} ,\omega +\omega ^{\prime} )\\  &  & \cdot \,{\Delta }_{1}^{R\,(+)}(p-l,\tilde{\omega }-\omega ^{\prime} )\,ig\,{}_{m}\langle \gamma (k^{\prime} )|\tilde{{\mathscr{U}}}(\omega ^{\prime} )|\gamma (l)\rangle _{s}\,ig\\  &  & \cdot \,{\Delta }_{2}^{R\,(+)}(p,\tilde{\omega })\tilde{J}(p,\tilde{\omega })\\  &  & \cdot \,{\Delta }_{1}^{R\,(+)}(k,\omega ){\Delta }_{1}^{R\,(-)}(k,{q^{\prime} }_{0}+\omega ),\end{array}\end{array}$$

27 $$\begin{array}{rcl} & = & {\int }^{}\,\frac{dp}{2\pi }\frac{d\omega ^{\prime} }{2\pi }{[|{J}_{0}|{\Delta }_{2}^{R(+)}(p,{\omega }_{e})]}^{2}\\  &  & \cdot \,{g}^{4}|\tilde{\Lambda }(\omega ^{\prime} ,y){|}^{2}\\  &  & \cdot \,{\Delta }_{1}^{R(+)}(p-{k}_{\omega ^{\prime} },{\omega }_{e}-\omega ^{\prime} )\\  &  & \cdot \,{e}^{2}{\lambda }^{2}\,{\int }^{}\,\frac{d\omega }{2\pi }\frac{dk}{2\pi }\,{k}^{2}\,{\Delta }_{1}^{R(+)}(k,\omega )\\  &  & \cdot \,{\Delta }_{2}^{R(+)}({k}_{\omega ^{\prime} }+k,\omega ^{\prime} +\omega )\\  &  & \cdot \,{\Delta }_{1}^{R(+)}(k,\omega )\,{\Delta }_{1}^{R(-)}(k,{q^{\prime} }_{0}+\omega ),\end{array}$$

where the superscript *R* represents the *retarded* propagators for the fields, and $\tilde{J}(p,\tilde{\omega })=2\pi {J}_{0}\,{e}^{ipNa/2}\,\delta (\tilde{\omega }-{\omega }_{e})$, or $J(x,t)={J}_{0}\,\delta (x+Na/2)\,{e}^{-i{\omega }_{e}t}$ which is a source of particle with energy ${\omega }_{e}$ located at the left end of the array $x=-\,Na/2$. Here we shall define a function $\zeta (\omega ^{\prime} ,{q^{\prime} }_{0})$ as,

28 $$\begin{array}{rcl}\zeta (\omega ^{\prime} ,{q^{\prime} }_{0}) & = & {\int }^{}\,\frac{dk}{2\pi }\frac{d\omega }{2\pi }\,{k}^{2}\,{\Delta }_{1}^{R(+)}(k,\omega )\,{\Delta }_{2}^{R(+)}({k}_{\omega ^{\prime} }+k,\omega ^{\prime} +\omega )\\  &  & \cdot \,{\Delta }_{1}^{R(+)}(k,\omega )\,{\Delta }_{1}^{R(-)}(k,{q^{\prime} }_{0}+\omega ),\\  &  & {\rm{and}}\,{-\frac{\partial }{\partial {q}_{0}}{\rm{Im}}\zeta (\omega ^{\prime} ,{q}_{0})|}_{{q}_{0}=0}\\  & \approx  & \frac{(1+\pi /4)\,{\pi }^{2}}{\sqrt{2}}\,\frac{1}{{a}^{3}\delta \lambda }\cdot {\rm{Re}}\,[\frac{1}{{(\omega ^{\prime} -\nu -\lambda \sin {k}_{\omega ^{\prime} }a+2i\delta )}^{2}}].\end{array}$$

Then the modification to the conductivity from another parallel array of 2LA is,

29 $$\begin{array}{rcl}\Delta {\sigma }_{DC}^{(P)}({\omega }_{e},y) & = & {-\frac{\partial }{\partial {q}_{0}}{\rm{Im}}{\langle j({q}_{0})j(-{q}_{0})\rangle }^{(P)}|}_{{q}_{0}=0}\\  & = & {e}^{2}{\lambda }^{2}{g}^{4}\,{\int }^{}\,\frac{dp}{2\pi }\frac{d\omega ^{\prime} }{2\pi }[{\Delta }_{2}^{R(+)}{(p,{\omega }_{e}]}^{2}\,{\Delta }_{1}^{R(+)}(p-{k}_{\omega ^{\prime} },{\omega }_{e}-\omega ^{\prime} )\cdot \\  &  & \cdot \,|{J}_{0}{|}^{2}\,|\tilde{\Lambda }(\omega ^{\prime} ,y){|}^{2}\cdot (\,-\,){\frac{\partial }{\partial {q}_{0}}{\rm{Im}}\zeta (\omega ^{\prime} ,{q}_{0})|}_{{q}_{0}=0}.\end{array}$$

If there is no external source, the measured array is independent of the source array, and its retarded current-current correlation and the DC conductivity for the measured array can be obtained through calculation similar to that done in ref.^58^,

30 $$\begin{array}{l}{\langle j({q^{\prime} }_{0})j(-{q^{\prime} }_{0})\rangle }^{(0)}\\ \begin{array}{rcl} & = & {e}^{2}{\lambda }^{2}\,{\int }^{}\,\frac{dk}{2\pi }\frac{d\omega }{2\pi }\frac{dl}{2\pi }\frac{dl^{\prime} }{2\pi }\,{k}^{2}\,{\Delta }_{1}^{R(+)}(k,\omega )\,{\Delta }_{1}^{R(-)}(k,{q^{\prime} }_{0}+\omega ),\\  &  & {\rm{and}},\,{\sigma }_{DC}^{(0)}={-\frac{\partial }{\partial {q}_{0}}{\rm{Im}}{\langle j({q}_{0})j(-{q}_{0})\rangle }^{(0)}|}_{{q}_{0}=0}=\frac{{\pi }^{2}}{32}\frac{{e}^{2}\lambda }{{a}^{3}\delta }.\end{array}\end{array}$$

**Figure 6:**
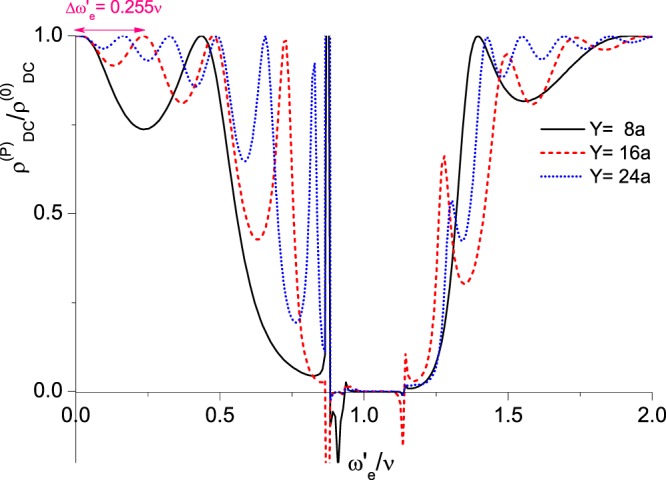
DC resistivity with input frequency. DC resistivity ${\rho }_{DC}^{(P)}={({\sigma }_{DC}^{(P)})}^{-1}$ of the measured array (in units of ${\rho }_{DC}^{(0)}$) versus ${\omega ^{\prime} }_{e}={\omega }_{e}-{\varepsilon ^{\prime} }_{1}$ (in units of $\nu$), the difference between the input frequency from the external source of the source array and the renormalized ground state energy. Here, we have plots for three different separations between the measured array and the source array at $Y=8a$, 16*a*, & 24*a*.

**Figure 7:**
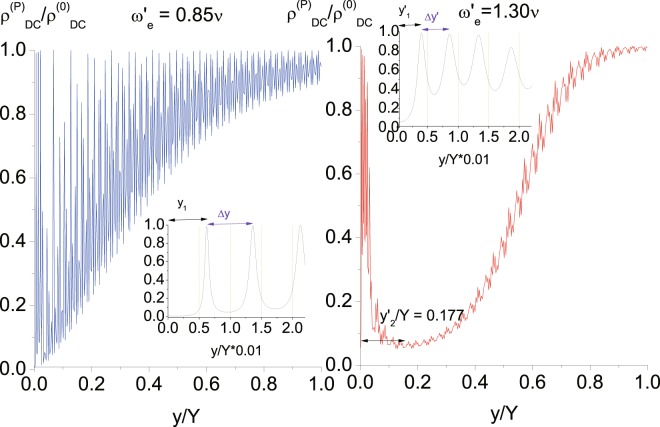
DC resistivity with separation. DC resistivity ${\rho }_{DC}^{(P)}={({\sigma }_{DC}^{(P)})}^{-1}$ (in units of ${\rho }_{DC}^{(0)}$) of the measured array versus the separation from the source array up to $Y=630a$. (**a**) Figure on the left (named as Fig. 7(a)) depicts the DC resistivity when the incoming frequency of the external source ${\omega }_{e}=0.85\nu +{\varepsilon ^{\prime} }_{1}$ (${\omega ^{\prime} }_{e}=0.85\nu$). (**b**) Figure on the right (named as Fig. 7(b)) depicts the DC resistivity when the incoming frequency of the external source ${\omega }_{e}=1.30\nu +{\varepsilon ^{\prime} }_{1}$ (${\omega ^{\prime} }_{e}=1.30\nu$). Both insets show the details of the two curves of resistivity at short separations up to 0.02*Y* = 12.6*a*, respectively.

The total DC conductivity ${\sigma }_{DC}^{(P)}$ is the sum of ${\sigma }_{DC}^{(0)}$ (Eq. (30)) & $\Delta {\sigma }_{DC}^{(P)}$ (Eq. (29)),

31 $${\sigma }_{DC}^{(P)}({\omega }_{e},y)={\sigma }_{DC}^{(0)}+\Delta {\sigma }_{DC}^{(P)}({\omega }_{e},y),$$

and the figures of ${({\sigma }_{DC}^{(P)})}^{-1}$ versus the source frequency ${\omega }_{e}$, and ${({\sigma }_{DC}^{(P)})}^{-1}$ versus the separation *y* between arrays, are depicted in Figs 6 and 7, respectively.

It is demonstrated in Fig. 6 that DC resistivity can reach zero; and in some regions, it can even be negative. The property of zero resistance has been found in many experiments in 2DEG systems irradiated by microwave on relatively pure samples^1,4,8–21^. And negative resistance arises in the experimental work of ref.^11^. We shall investigate the diverse behaviors of resistivity in our system shown in Figs 6 and 7 in more details in the Discussion section.

## Discussion

For convenience, we shall define a parameter ${\omega ^{\prime} }_{e}$ which is the energy difference between the external frequency and the renormalized ground state energy, ${\omega ^{\prime} }_{e}\equiv {\omega }_{e}-{\varepsilon ^{\prime} }_{1}$. Then in Eq. (29), it can be seen from ${\Delta }_{1}^{R(+)}(p-{k}_{\omega ^{\prime} },{\omega }_{e}-\omega ^{\prime} )$ (Eq. (16)) that most of the contributions in the integration over the photonic energy $\omega ^{\prime}$ are from the region ${\omega ^{\prime} }_{e}\lesssim \omega ^{\prime} \lesssim {\omega ^{\prime} }_{e}+2\lambda$. And seeing from ${[{\Delta }_{2}^{R(+)}(p,{\omega }_{e})]}^{2}$ in Eq. (29), when ${\omega }_{e}$ is around ${\varepsilon ^{\prime} }_{2}$, or ${\omega ^{\prime} }_{e}$ is around $\nu ={\varepsilon ^{\prime} }_{2}-{\varepsilon ^{\prime} }_{1}$, the modification to the conductivity $\Delta {\sigma }_{DC}^{(P)}({\omega }_{e},y)$ becomes its maximum. Thus, the parameter ${\omega ^{\prime} }_{e}(\,=\,{\omega }_{e}-{\varepsilon ^{\prime} }_{1})$ is quite important for the behaviors of the amplitude of the emitted photon $\tilde{\Lambda }(\omega ^{\prime} ,y)$ (Eq. (24)) and the conductivity of the measured array ${\sigma }_{DC}^{(P)}({\omega }_{e},y)$ (Eq. (31)).

In the following subsections, we shall study the behaviors of the resistivity of the measured array vs. the external frequency ${\omega }_{e}$ (or ${\omega ^{\prime} }_{e}$), and separations between arrays, in addition to phenomena of zero & negative resistivity shown in Fig. 6. They can be understood by investigating $|\tilde{\Lambda }(\omega ^{\prime} ,y){|}^{2}$ (Eq. (24)) which is the probability of photon emitted from the source array to the measured array, and ${-\frac{\partial }{\partial {q}_{0}}{\rm{Im}}\zeta (\omega ^{\prime} ,{q}_{0})|}_{{q}_{0}=0}$ (Eq. (28)) which characterizes the conductivity of the measured array through the photonic dispersion relation; both of them appear in Eq. (29) of $\Delta {\sigma }_{DC}^{(P)}({\omega }_{e},y)$.

### Behaviors of resistivity vs. the external frequency *ω*_*e*_

The behaviors of the resistivity of the measured array with respect to the incoming frequency ${\omega }_{e}(\,=\,{\omega ^{\prime} }_{e}+{\varepsilon ^{\prime} }_{1})$ of the source array at three distances of separation $Y=8a$, 16*a*, & 24*a* are shown in Fig. 6.

1. As the input frequency of the external source is small (${\omega ^{\prime} }_{e}\gtrsim 0$), $|\tilde{\Lambda }(\omega ^{\prime} ,y){|}^{2}\sim 0$, and there are not many photons emitted from the source array. Therefore, the resistivity of the measured array is almost not changed as if it is alone.
2. As $0 < {\omega ^{\prime} }_{e}\ll \nu$, $|\tilde{\Lambda }(\omega ^{\prime} ,y){|}^{2}\sim {\cos }^{2}({\omega ^{\prime} }_{e}\,y+\varphi )$ with $\varphi$ nearly a constant, the periodic oscillations of the resistivity are from occurrences of standing waves, and therefore, the separation between peaks of ${\omega ^{\prime} }_{e}$ satisfies $(\Delta {\omega ^{\prime} }_{e}/c)\cdot y\sim \pi$. For $Y=16a$, we have $\Delta {\omega ^{\prime} }_{e}=\Delta {\omega }_{e}\sim 0.255\nu$.
3. As ${\omega ^{\prime} }_{e}\sim \nu$, due to large DOS around the energy gap shown in Fig. 4, many photons with energy close to the difference between the excited state energy and the ground state energy ($\nu$) are emitted from the source array. In the measured array, originally most atoms are in the (renormalized) ground state at zero temperature. Being almost fully occupied, ground state electrons can hardly hop to their neighbors, and the conductivity is poor. Once photons with frequency near $\nu$ emitted from the source array are absorbed in the measured array, electrons can be raised to the (renormalized) excited state. They whence can easily hop to their neighbors and transport. Furthermore, excited electrons are boosted by the absorbed photons with additional momentum ${k}_{x}={k}_{{\omega ^{\prime} }_{e}}$. As a result, the electron conductivity is significantly modified, and zero resistance appears^58^.
  The factor ${-\frac{\partial }{\partial {q}_{0}}{\rm{Im}}\zeta (\omega ^{\prime} ,{q}_{0})|}_{{q}_{0}=0}$ (Eq. (28)) appearing in $\Delta {\sigma }_{DC}^{(P)}$ (Eq. (29)) carries the information of zero and negative resistance of the measured array. Analytically, it is shown in Fig. 4 that ${K}_{\omega ^{\prime} }$ is real when $\omega ^{\prime}$ proceeds toward but not very near $\nu$. For some *λ*, $\omega ^{\prime} -\nu -\lambda \,\sin \,{k}_{{\omega }_{s}}a\sim O[\delta ]$; thus the denominator in the factor ${-\frac{\partial }{\partial {q}_{0}}{\rm{Im}}\zeta (\omega ^{\prime} ,{q}_{0})|}_{{q}_{0}=0}$ (Eq. (28)) would be very small and $\Delta {\sigma }_{DC}^{(P)}$ in Eq. (29) is of order ${\mathscr{O}}[{\sigma }_{DC}^{(0)}\cdot {g}^{4}|{J}_{0}{|}^{2}{\delta }^{-2}]$. For very small *δ*, the DC conductivity of the measured array would become very large, and meanwhile, the resistance goes to zero (Fig. 6).
4. For negative resistivity around $\omega ^{\prime} \sim \nu$, the momentum ${K}_{\omega ^{\prime} }$ of the corresponding photon is complex (Fig. 4) and the light wave is attenuated. Taking ${k}_{\omega ^{\prime} }\equiv {\rm{Re}}\,{K}_{\omega ^{\prime} }$ & ${\kappa }_{\omega ^{\prime} }\equiv {\rm{Im}}\,{K}_{\omega ^{\prime} }$, the real part of the square bracket in Eq. (28) is

32 $${\rm{Re}}\,[\cdots ]=\frac{{(\omega ^{\prime} -\nu -\lambda \sin {k}_{\omega ^{\prime} }a\cosh {\kappa }_{\omega ^{\prime} }a)}^{2}-{(\lambda \cos {k}_{\omega ^{\prime} }a\sinh {\kappa }_{\omega ^{\prime} }a+2\delta )}^{2}}{{[{(\omega ^{\prime} -\nu -\lambda \sin {k}_{\omega ^{\prime} }a\cosh {\kappa }_{\omega ^{\prime} }a)}^{2}+{(\lambda \cos {k}_{\omega ^{\prime} }a\sinh {\kappa }_{\omega ^{\prime} }a+2\delta )}^{2}]}^{2}}.$$

When $\omega ^{\prime} \sim \nu$, for some *λ*, the RHS of Eq. (32) becomes negative. For example, when parameters take values shown in Fig. 4, as ${\omega ^{\prime} }_{e}=1.114\,\nu$ & $\omega ^{\prime} \sim {\omega ^{\prime} }_{e}$, the above numerator is negative and the corresponding resistivity of the measured array is ${\rho }_{DC}^{(P)}=-\,0.219\,{\rho }_{DC}^{(0)}$ (Fig. 6). From a physical point of view, briefly speaking, the appearance of negative conductivity is related to the Bloch wave functions of electrons and photons. For an electron with momentum $k > 0$ transmitting in a lattice and interacting with photons on lattice sites, its eigenfunction is a Bloch wave function which is a linear combination of plane waves with momenta $k+2\pi n/a$’s, for all integer values of *n*. Among these plane waves, the $n=0$ & the $n=-\,1$ components dominate and represent the principal forward & backward scatterings, respectively^72,73^. As the energy of the dressed photon is around the energy gap ($\omega ^{\prime} \sim \nu$), under certain circumstances (as we illustrated above), the amplitude of the $n=0$ component of the Bloch wave of the electron being excited by absorbing a dressed photon with frequency $\omega ^{\prime}$ is small^73^, and the forward scattering diminishes. As a result, the backward scattering dominates and negative conductivity appears.

### Behaviors of resistivity vs. the separation between two arrays from 0 to *Y*

In Fig. 7(a), as the input frequency ${\omega ^{\prime} }_{e} < \nu$, the average of the resistivity increases monotonically with the separation between arrays (*y*). The periodic oscillations of the resistivity are from occurrences of standing waves, and therefore, the separation between peaks Δ*y* satisfies $\frac{{\omega ^{\prime} }_{e}}{c}\,\Delta y\sim \pi$. The first peak occurs approximately at $\frac{{\omega ^{\prime} }_{e}}{2c}\cdot {y}_{1}\sim \frac{\pi }{2}$ such that, roughly speaking, the amplitude of photon emitted from the source array to the measured array $\tilde{\Lambda }({\omega ^{\prime} }_{e},{y}_{1})$ (Eq. (24)) contributed by half of the *k*_*y*_’s within $[0,{\omega ^{\prime} }_{e}]$ is out of phase with that from the other half. For ${\omega ^{\prime} }_{e}=0.85\nu$ and $Y=630a$, we have ${y}_{1}\sim 4.725\,a=0.0075\,Y$, and $\Delta y\sim 4.725\,a=0.0075\,Y$ as are presented in the inset of Fig. 7(a).

For ${\omega ^{\prime} }_{e}\ge \nu$, the resistivity is shown in Fig. 7(b), and there are periodic oscillations due to standing wave as before. Nevertheless, the average of the resistivity goes up and down before it increases monotonically with the separation between arrays. This is originated from the energy gap (in the photonic dispersion relation) around where the DOS dominates (Fig. 4). The photonic energy satisfies $\omega {^{\prime} }^{2}\sim {({\omega ^{\prime} }_{e})}^{2}={\omega }_{{k}_{x}}^{2}+{k}_{y}^{2}$ where ${\omega }_{{k}_{x}}^{2}$ & ${k}_{y}^{2}$ are the (kinetic) energies associated with the *x*-momentum *k*_*x*_ & the *y*-momentum *k*_*y*_ of the emitted photon, respectively. As the photonic frequency $\omega ^{\prime} \sim {\omega ^{\prime} }_{e}\ge \nu$, ${\omega }_{{k}_{x}}$ can be larger than the lower edge of the energy gap for small *k*_*y*_. Most of the contributions to the amplitude of the emitted photon $\tilde{\Lambda }({\omega ^{\prime} }_{e},y)$ are from photons with ${\omega }_{{k}_{x}}$ close to the energy gap, *i*.*e*., ${k}_{x}\sim {k}_{x0}$ & ${\omega }_{{k}_{x}}\sim {\omega }_{{k}_{x0}}=\nu$ with a spreading $\delta {\omega }_{{k}_{x0}}$ of the energy gap around which the DOS dominates^71^. As ${k}_{x}\sim {k}_{x0}$, we have ${k}_{y}\sim {k}_{y0}=\sqrt{{({\omega ^{\prime} }_{e})}^{2}-{\omega }_{{k}_{x0}}^{2}}=\sqrt{{({\omega ^{\prime} }_{e})}^{2}-{\nu }^{2}}$. In terms of the parameters listed in Fig. 4, the spreading $\delta {\omega }_{{k}_{x0}}\sim 0.03\nu$ (see Fig. 4). Accordingly, we can then understand behaviors of the resistivity with respect to the separation between arrays as are shown in Fig. 7(b) through the following discussions.

1. For those photons with *y*-momentum ${k}_{y}^{(-)}\lesssim {k}_{y0}$ (${k}_{y}^{(+)}\gtrsim {k}_{y0}$), they carry kinetic energy of the *x*-momentum ${\omega }_{{k}_{x}}\gtrsim \nu$ (${\omega }_{{k}_{x}}\lesssim \nu$), and are on the right (left) edge of the gap. As $y={y^{\prime} }_{1}$ & ${k}_{y0}\,{y^{\prime} }_{1}\sim \pi /2$, we have $\cos ({k}_{y}^{(-)}{y^{\prime} }_{1}) > 0$, & $\cos ({k}_{y0}^{(+)}{y^{\prime} }_{1}) < 0$ in $\tilde{\Lambda }(\omega ^{\prime} ,y)$ (Eq. (24)). Therefore, those photons around the left edge of the energy gap are out of phase with those around the right. This would reduce to the most extent the amplitude of the emitted photon to the measured array. Then we have the location of the first peak of the average of the resistivity at $y={y^{\prime} }_{1}\sim \pi /2/{k}_{y0}\sim \pi /2/\sqrt{{({\omega ^{\prime} }_{e})}^{2}-{\nu }^{2}}$. And the separation between peaks Δ*y*′ satisfies ${\omega ^{\prime} }_{e}\,\Delta y^{\prime} \sim \pi$, as we explained earlier for ${\omega ^{\prime} }_{e}=0.85\nu$. It follows that ${y^{\prime} }_{1}\sim 2.4\,a=0.0038\,Y$, & $\Delta y^{\prime} \sim 3.1\,a=0.0049\,Y$, for ${\omega ^{\prime} }_{e}=1.30\,\nu$ & $Y=630\,a$ as are shown in the inset of Fig. 7(b).
2. As the separation *y* between the two arrays increases from ${y^{\prime} }_{1}$, not all photons around the left edge of the energy gap are out of phase with those around the right; and the amplitude of the emitted photon to the measured array grows. Therefore, the average of the resistivity decreases.
3. As the separation *y* between the two arrays increases to ${y^{\prime} }_{2}$, the average of the resistivity decreases to its minimum. This can be understood by looking at the phases of those photons around the energy gap at ${\omega }_{{k}_{x0}}(\,=\,\nu )$. For emitted photons carrying *x*-momentum ${k}_{x}\sim {k}_{x0}$, or equivalently ${k}_{y}\sim {k}_{y0}=\sqrt{{({\omega ^{\prime} }_{e})}^{2}-{\omega }_{{k}_{x0}}^{2}}$, they have ${\omega }_{{k}_{x}}\sim {\omega }_{{k}_{x0}}=\nu$ and are around the energy gap. At the distance $y={y^{\prime} }_{2}$, those emitted photons with ${\omega }_{{k}_{x}}$ located within the spreading of the energy gap are all in phase with each other, *i*.*e*., $\cos \,[({k}_{y0}-\delta {k}_{y0}/2){y^{\prime} }_{2}]$ & $\cos \,[({k}_{y0}+\delta {k}_{y0}/2){y^{\prime} }_{2}]$ are of the same sign in $\tilde{\Lambda }(\omega ^{\prime} ,y)$ (Eq. (24)). It follows that ${k}_{y0}\cdot {y^{\prime} }_{2}\sim n\pi$, and $\delta {k}_{y0}\cdot {y^{\prime} }_{2}\sim \pi$. To find the spreading $\delta {k}_{y0}$ around ${k}_{y0}$ so as to get ${y^{\prime} }_{2}$, we have the spreading $\delta {\omega }_{{k}_{x0}}$ around the energy gap ${\omega }_{{k}_{x0}}=\nu$ as
  33 $$\delta {\omega }_{{k}_{x0}}\sim {\delta \sqrt{{({\omega ^{\prime} }_{e})}^{2}-{k}_{y}^{2}}|}_{y\sim {y}_{0}}=\frac{{k}_{y0}\,\delta {k}_{y0}}{\sqrt{{({\omega ^{\prime} }_{e})}^{2}-{k}_{y0}^{2}}}=\frac{\sqrt{{({\omega ^{\prime} }_{e})}^{2}-{\nu }^{2}}}{\nu }\cdot \delta {k}_{y0}.$$
  Thus, we have
  34 $${y^{\prime} }_{2}\sim \frac{\pi }{\delta {k}_{y0}}\sim \frac{\pi \sqrt{{({\omega ^{\prime} }_{e})}^{2}-{\nu }^{2}}}{\nu \cdot \delta {\omega }_{{k}_{x0}}},$$
  35 $$\begin{array}{ll}{\rm{and}} & {y^{\prime} }_{2}\sim 111\,a=0.177\,Y,\\ {\rm{for}} & {\omega ^{\prime} }_{e}=1.30\nu ,Y=630\,a,\,\& \,\delta {\omega }_{{k}_{x0}}\sim 0.03\nu ,\end{array}$$
  as is shown in Fig. 7(b).
4. As the separation *y* between the two arrays increases from ${y^{\prime} }_{2}$ and further, more photons around the spreading of the energy gap (or around ${k}_{y0}$) get out of phase with each other, and the resistivity increases.

In summary, we explored interactions between two (parallel) arrays of 2LA through emitting and absorbing photons via QED interaction. We calculate the *t*-matrix of the two fields, upper & lower fields, for electrons. The *t*-matrix summarizes the ladder diagrams of binary collisions between upper & lower fields interacting with each other through hard-core interaction. We take the *t*-matrix at low energy as the (finite) effective coupling between upper & lower fields. And we find renormalized propagators of the upper & lower fields. Their corresponding renormalized eigenkets are orthogonal to each other. Then we include in our calculations the interactions between photons and electrons through diagrammatic techniques in terms of renormalized propagators.

Due to transportation with repeated scatterings in the source array which is a linear lattice of 2LA, the emitted photons are Bloch waves^58^ with a nonlinear dispersion relation which has a gap around the spacing between 2LA energy levels. This significantly modifies the group velocity and the DOS of the photonic field. In addition, standing waves can occur for photonic Bloch wave as it propagates from the source to the measured array. It follows that the conduction behaviors of the measured array can be very diversified according to the input frequency of the source and the separation between arrays. As a result, the resistivity of the measured array can be zero or negative, and can also show oscillations when we change the incoming frequency of the source array and the separation between arrays.

The theoretical scheme that we investigated in this work can be experimentally realized in many two-level-system arrays such as superconducting-qubit array^74–77^, trapped atom array^59–63^, and gate-control dot array^78^. For example, the source array can be achieved experimentally in the semiconductor quantum-dot array^78^. By connecting to the source and drain reservoirs, the electron in the source-reservoir with energy around (renormalized) excited energy of the array can transport through the quantum-dot array to form a source array. The energy of the electron tunneling out can be further tuned by changing the applied bias voltage^79^ between source and drain reservoirs. The scheme has also the potential for measuring the photoresisitance version of the quantum interference, such as super-radiance^80^, the quantum phase transition^81^, and the optical non-linearity^82–84^. Furthermore, the scheme can be applied for reading out the quantum memory^85^.

## Methods

We define the Hamiltonian of a quantized photonic field in Eq. (2). Then a Hamiltonian of arrayed 2LA with hopping term is introduced in Eq. (4). And Eq. (5) represents a hard-core interaction between the excited electron and the electron in the ground state to assure that only one electron can be present in an atom. The Hamiltonian ${ {\mathcal H} }_{int}(A,\{c\})$ describing the interaction between *bare* photons and *bare* electrons is introduced in Eq. (7). We then obtain an effective interaction ${H}_{eff}^{(4)}({c}^{\dagger },c)$ (Eq. (11)) with an effective coupling which includes all the repeated scatterings between the excited electron and the ground state electron with the hard-core interaction in the subsection of *t*-matrix. Thereafter, we calculate the (renormalized) propagators of electrons with the modifications by the self-masses from the effective interaction ${H}_{eff}^{(4)}({c}^{\dagger },c)$. Then, by the Dyson’s equation (Eq. (17)), we obtain the propagator of the *dressed* photon $\tilde{G}(k,\omega ;k^{\prime} ,\omega ^{\prime} )$ which includes the interactions between the photons and electrons. The function $\tilde{\Lambda }(\omega ^{\prime} ,y)$ is then introduced, and its Fourier transform in time *t*′ describes the amplitude of one photon in the source array propagating to the measured array with separation *y* in time interval *t*′. Finally, we calculate the DC conductivity by the Kubo formula in Eq. (25).

As for the simulation process, we consider two different situations, one with fixed separation between arrays and varying frequency of the source, and the other is done with fixed frequency of the source and varying separation between arrays. Then we calculate the total resistivity numerically under the two above situations with specific parameters. The results are presented in the Discussion subsection.
